# Amino-Acid-Conjugated Natural Compounds: Aims, Designs and Results

**DOI:** 10.3390/molecules27217631

**Published:** 2022-11-07

**Authors:** Hanggara Arifian, Rani Maharani, Sandra Megantara, Amirah Mohd Gazzali, Muchtaridi Muchtaridi

**Affiliations:** 1 Department of Pharmaceutical Analysis and Medicinal Chemistry, Faculty of Pharmacy, Universitas Padjadjaran, Sumedang 45363, Indonesia; 2 Department of Pharmacochemistry, Faculty of Pharmacy, Universitas Mulawarman, Samarinda 75119, Indonesia; 3 Research Collaboration Centre for Theranostic Radiopharmaceuticals, National Research and Innovation Agency (BRIN), Jakarta 10340, Indonesia; 4 Department of Chemistry, Faculty of Mathematics and Natural Sciences, Universitas Padjadjaran, Jatinangor 45363, Indonesia; 5 School of Pharmaceutical Sciences, Universiti Saisn Malaysia, Penang 11800, Malaysia

**Keywords:** natural compounds, amino acid, conjugation, chemical synthesis

## Abstract

Protein is one of the essential macronutrients required by all living things. The breakdown of protein produces monomers known as amino acids. The concept of conjugating natural compounds with amino acids for therapeutic applications emerged from the fact that amino acids are important building blocks of life and are abundantly available; thus, a greater shift can result in structural modification, since amino acids contain a variety of sidechains. This review discusses the data available on amino acid–natural compound conjugates that were reported with respect to their backgrounds, the synthetic approach and their bioactivity. Several amino acid–natural compound conjugates have shown enhanced pharmacokinetic characteristics, including absorption and distribution properties, reduced toxicity and increased physiological effects. This approach could offer a potentially effective system of drug discovery that can enable the development of pharmacologically active and pharmacokinetically acceptable molecules.

## 1. Introduction

With the passage of time, the need for more effective therapeutics is increasing, with more diseases being discovered and more health-related problems being understood through advanced research and studies [1]. Often, natural resources from plants, animals and microorganisms are described as suitable candidates [2], and many established medicines originating from natural products were inspired by the chemical synthesis of biologically active materials from natural products. The molecular framework of natural compound synthesis is recognised as an abundant resource for medicinal chemistry and drug development [3,4,5].

Not all natural products are unsuitable for use as medications [6,7,8]. Some of them may be considered as potentially useful compounds if they are proven to have certain favourable pharmacological and pharmacokinetic characteristics [9,10]. Many natural compounds, however, require structural modifications through chemical synthesis approaches to meet the required criteria of effective and safe therapeutics [11,12,13].

Conjugation with amino acids is one of the means of remodelling the structure of natural compounds [14]. Amino acids are chosen based on the premise that they are the most quintessential monomers of living systems [15,16]. Amino acids are also the primary construction blocks for proteins, as well as the substance that sustains biological activities, in addition to playing integral roles in living organisms [17,18]. Moreover, as amino acids have various sidechains, structural alteration is attainable, with a broader shift [19].

The manipulation of amino acid is relatively simple, with a myriad of possible targeted pharmacological activities. To date, several amino acid–natural compound conjugates have been reported that involve curcumin, astaxanthin and quercetin, among others [20,21,22]. The conjugations aimed to enhance the pharmacological activities, lessen the toxicity, improve the target specificity and increase absorption via peptide transporters [23,24,25].

This review examines the available literature on amino acid–natural compound conjugations. The method of conjugation is discussed and analysed to gain insightful information on the production of amino acid–natural compound conjugates for future medical applications.

## 2. Natural Compounds Conjugated with Amino Acids

Various natural compounds have undergone structural modifications and been evaluated as potential therapeutics. This review explores all the relevant studies on this subject, with a specific interest in semisynthetic modifications of the natural compounds. The purpose, synthetic strategies and biological outcomes are also discussed.

### 2.1. Alkaloids

Alkaloids have a wide range of structural variations. The presence of a basic nitrogen atom is the common denominator. The nitrogen can be a primary, secondary or tertiary amine [26]. Analgesic (codeine), central nervous system depressant (morphine), anti-hypotensive (ephedrine), anticholinergic (atropine), antiemetic (scopolamine) and antimalarial (quinine) products are among the examples of natural-based pharmacological agents in this class [27].

However, alkaloids have unfavourable physical and chemical characteristics, such as a low solubility, low stability at physiological pH, low oral absorption and low overall bioavailability, in addition to having a quick clearance from the body. These problems reduce the efficacies of alkaloids [28,29]. To solve these issues, a number of alkaloid derivatives conjugated with amino acids have been developed, such as piperine, camptothecin and quinine.

#### 2.1.1. Piperine

**Figure 1:**
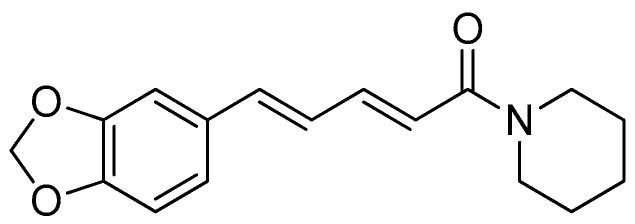
Piperine.

Piperine (Figure 1) is an alkaloid found in various *Piperaceae* family, including *Piper nigrum*, *Piper longum*, *Piper chaba*, *Piper guineense* and *Piper sarmentosum* [30,31]. Piperine is responsible for the peculiar biting feeling associated with black pepper. Black pepper is traditionally used in therapeutics and preservatives and for its scent, in addition to being widely used in human diets [32,33].

The synthesis of amino acid–piperine conjugates has been described as having the potential to produce antileishmanial [34] and anticancer agents [35]. As piperine is naturally abundant, this compound is a favourable choice for manipulation via this approach. Furthermore, the chemical reactions and processes involved are simple and straightforward, making the scaled-up manufacturing of piperine derivatives highly possible [35].

The inhibitory activity and the mode of action of piperine against *Leishmania donovani* [36] can be enhanced if the piperine is in the form of oil-in-water emulsion [37] or mannose-coated liposomes [38]. In addition, amino acid esters have also demonstrated inhibitory actions against *Leishmania amazonensis* and *Leishmania mexicana* following their accumulation in the phagolysosomes of these parasites [39].

**Scheme 1:**
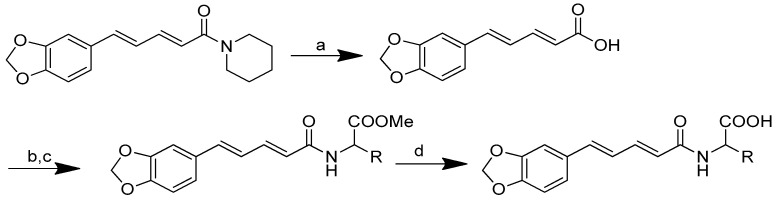
Reagents and conditions: (**a**) 20% KOH, CH_3_OH, 80°C, 3 days, 88%; (**b**) CH_3_SO_2_Cl, Et_3_N, DCM, 0 °C, 30 min, 85%; (**c**) amino acid methyl ester, Et_3_N, DCM, RT, 40–75%; (**d**) KF-Al_2_O_3_ (40%), microwave, 1000 W, 3–4 min, 70–80%. R in order: L-phenylalanine; L-tyrosine; L-valine; L-methionine; L-tryptophan.

**Scheme 2:**
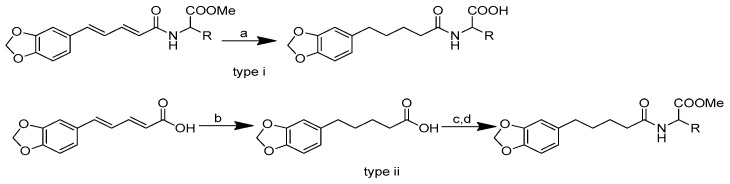
Reagents and conditions: type i (**a**) 5% Pd/C, H_2_ (40 Psi), MeOH, 30 min; type ii (**b**) 5% Pd/C, H_2_ (40 Psi), MeOH, 30 min; (**c**) CH_3_SO_2_Cl, Et_3_N, DCM, 0 °C, 30 min, 85%; (**d**) amino acid methyl ester, Et_3_N, DCM, RT.

The synthesis of amino acid–piperine conjugate was divided into three series (Scheme 1). First of all, piperine was converted to piperic acid via the hydrolysis of its amide bond. The carboxyl group in piperic acid provides a place for the formation of a covalent bond with the amino acids through the formation of a new amide bond. Piperic acid was then conjugated to a protected amino acid using menthane-sulfonyl chloride, CH_3_SO_2_Cl, in CH_2_Cl_2_ at 0 °C to produce piperoyl–amino acid methyl ester conjugates. The desired piperoyl–amino acid methyl ester conjugates (**3**–**7**) were reported to be in the range of 40–75% yields. Later, deprotection was conducted using Al_2_O_3_ in microwave-assisted solid phase until the ester group was converted into free carboxyl groups (**8**–**12**) with 70–80% yields. The other analogues are the saturated derivatives of the conjugate of the first series. The approach used for the synthesis of these analogues was carried out in two ways, namely, (i) the direct hydrogenation of the first series’ conjugate, and (ii) the initial hydrogenation of piperic acid to give tetrahydropiperic acid, followed by the conjugation of the saturated piperic acid with amino acid methyl esters [34] (Scheme 2).

All of the conjugates showed potential against both the amastigote and the promastigote forms of the parasite (Table 1). The IC_50_ values of piperine against the amastigotes and promastigotes were 0.7 and 2.5 mM, respectively. When compared with piperine or amino acid esters, the piperoyl–amino acid ester conjugates (**3**–**7**) exhibited a significant increase in their activity.

**Figure 2:**
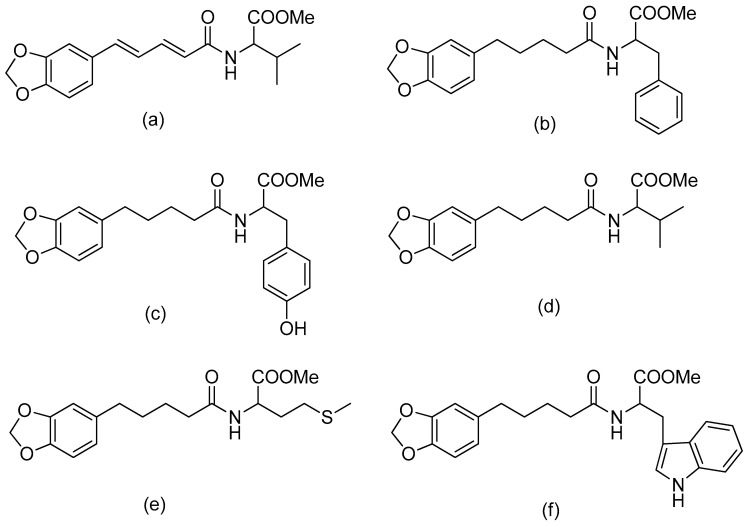
(**a**) Piperoyl-L-valine methyl ester; (**b**) tetrahydropiperoyl-L-phenylalanine methyl ester; (**c**) tetrahydropiperoyl-L-tyrosine methyl ester; (**d**) tetrahydropiperoyl-L-valine methyl ester; (**e**) tetrahydropiperoyl-L-methionine methyl ester; (**f**) tetrahydropiperoyl-L-tryptophan methyl ester.

The combination of piperic acid and valine methyl ester (Figure 2) was shown to be the most efficient against amastigotes, with an IC_50_ value of 0.07 mM (compound **5**) [34]. The effectiveness of valine may be related to the strong need of valine to produce NADH in the procyclic phase of *Leishmania donovani* [40]. On the other hand, the antileishmanial activity against amastigotes was reduced when the carboxyl group of amino acid functionality was deprotected in compounds **8**–**12** (Table 1) compared to compounds **3**–**7** (with the protected amino acid group). The antileishmanial activity of the conjugates **13**–**17** against the amastigotes decreased when the piperine subunit’s conjugated double bonds were reduced. However, the antileishmanial activity of the conjugates against promastigotes was boosted. The most active compound against the promastigotes was tetrahydropiperoyl tryptophan methyl ester (compound **17**), with an IC_50_ value of 0.47 mM.

#### 2.1.2. Camptothecin

**Figure 3:**
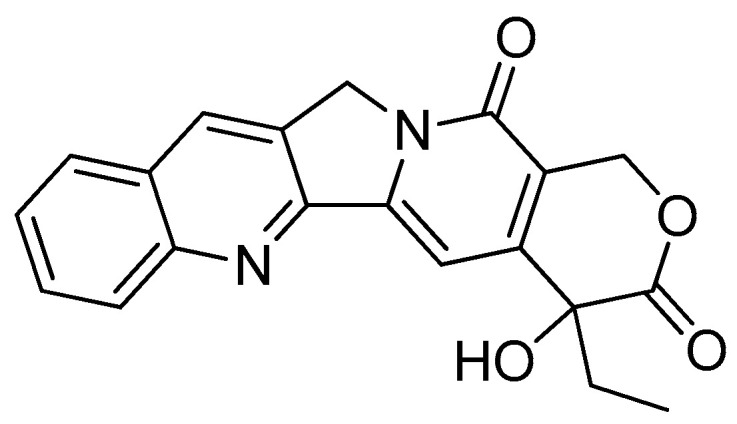
Camptothecin.

Camptothecin (Figure 3), a quinolone type of alkaloid, is a potent antitumor agent isolated from *Camptotheca acuminate* [41,42]. However, it has a low solubility and some adverse effects [43,44]. One of the strategies used to overcome this problem is the conjugation of camptothecin with poly-α-L-glutamic acid [45].

**Scheme 3:**
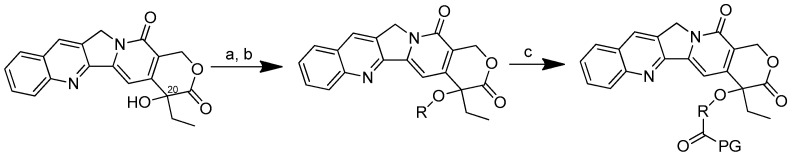
Synthesis of the PG conjugates of S-camptothecin. Reagents and conditions: (**a**) amino acid linker (R), DIPC, DMAP, DMF; (**b**) 50% TFA-CH_2_Cl_2_; (**c**) poly-R-(L-glutamic acid) (PG), DIPC, DMAP, DMF. R is the following amino acids: conjugate no. (**2**): L-glycine; (**3**) L-alanine; (**4**) β-alanine; (**5**) 4-NH-butyryl; (**6**) 2-O-acetyl; (**7**) 4-O-butyryl; (**8**) γ-glutamic acid.

The conjugation of 50 kDa poly-R-(L-glutamic acid) (PG) and L-camptothecin was achieved by means of the esterification of the hydroxyl group at the C-20 position with amino acid as a linker (Scheme 3). Amino acid was added to a solution of camptothecin, DMAP and DIPC in DMF at room temperature to form amino acid–camptothecin. Then, 50% TFA was used to deprotect the amino acid linker. Thereafter, an amino-acid-linked camptothecin solution in anhydrous DMF was added to the poly-R-(L-glutamic acid) solution. The DMAP and DIPC in DMF were gradually added to the combination and chilled in an ice bath. The mixture was stirred for two days at room temperature [45].

**Figure 4:**
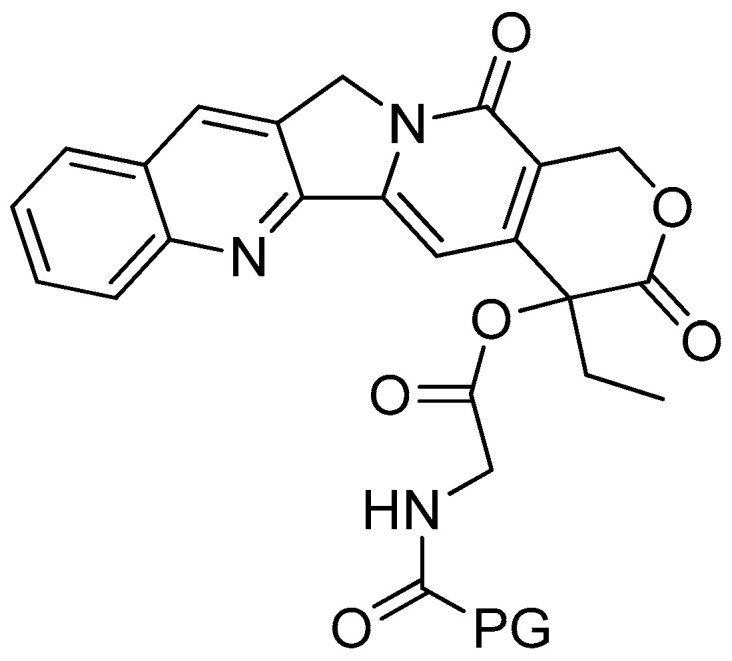
Poly-R-(L-glutamic acid)-L-glycine-camptothecin.

The in vivo activity of the conjugates was evaluated against B-16 melanomas (Table 2). When compared to a similar camptothecin dose level, poly-*R*-(L-glutamic acid)-glycine-camptothecin (Figure 4) was found to have the best antitumor efficacy. This conjugate was able to suppress the growth of B-16 tumour cells following 48 h of treatment at a lower dose in comparison to the poly-*R*-(L-glutamic acid)-camptothecin and the other poly-*R*-(L-glutamic acid)-linker-camptothecin conjugates.

#### 2.1.3. Quinine

**Figure 5:**
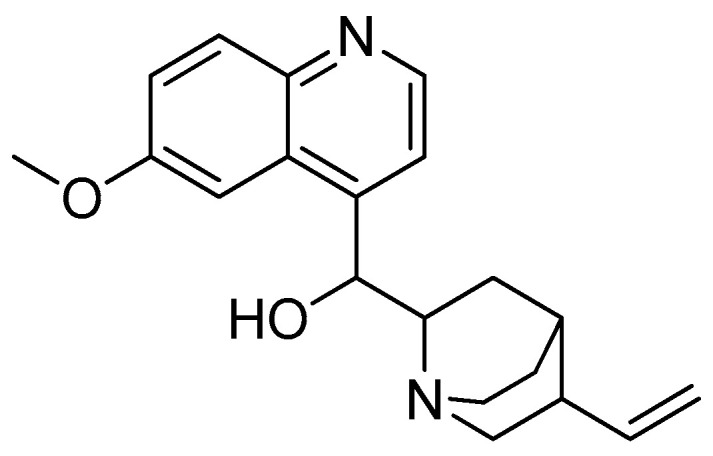
Quinine.

Quinine (Figure 5) was isolated from *Cinchona* bark for the first time in France in 1820, and it was recognised as a major component of the bark [46,47]. Given that most antimalarial medications reveal parasite resistance within a few years after their introduction to the market, quinine has a relatively stronger track record [48,49]. A low parasite resistance towards quinine has made the structure favourable for overcoming infections by this parasite [50].

Amino acid conjugates of quinine were synthesised to produce new compounds using a different synthetic strategy from the development of the previous quinine derivatives, such as chloroquine. The conjugates’ synthesis also aimed to overcome the resistance problems, increase the drug delivery efficiency and improve the performance of quinine as an antimalarial agent [51,52].

**Scheme 4:**
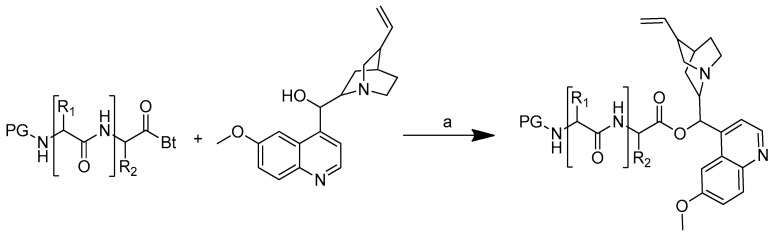
Synthesis of quinine conjugates. PG: protected groups. (**a**): K_2_CO_3_, DMF. R is the following amino acids: conjugate no. (**2**) R_1_: L-glycine; (**3**) R_1_: L-alanine; (**4**) R_1_: L-phenylalanine; (**5**) R_1_: L-isoleucine; (**6**) R_1_: L-histidine; (**7**) R_1_: L-serine; (**8**) R_1_: L-glutamic acid; (**9**) R_1_: L-lysine; (**10**) R_1_: L-aspartic acid; (**11**) R_1_: L-cysteine; (**12**) R_1_: L-alanine, R_2_: L-phenylalanine; (**13**) R_1_: L-valine, R_2_: L-leucine; (**14**) R_1_: L-isoleucine, R_2_: L-glycine.

The synthetic strategy used was to form an ester bond between the quinine and amino acid or peptide. The amino acid or peptide was selected based on the difference in the amino acid polarity. Quinine was *O*-acylated with *N*-protected acylbenzotriazoles in the presence of anhydrous potassium carbonate in anhydrous DMF for 10 to 30 min at 50 °C to produce amino acid and peptide conjugates of quinine (Scheme 4). The reaction time was shortened by utilising microwave irradiation, which is important for minimising the formation of epimers during the conjugation process [51].

**Figure 6:**
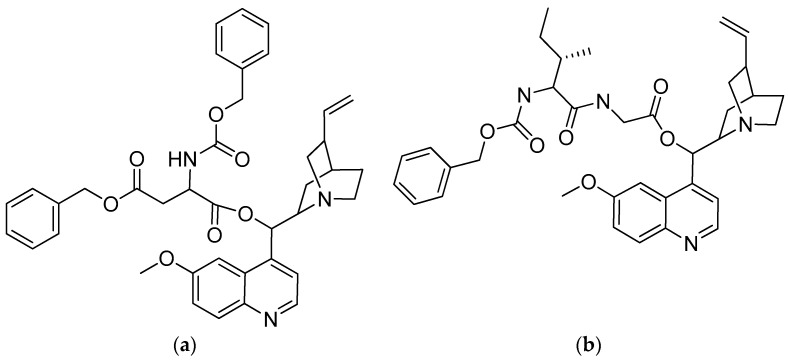
(**a**) Acylbenzotriazoles-L-aspartic acid-quinine; (**b**) acylbenzotriazoles-L-isoleucine-glycine-quinine.

In vitro tests were performed on the quinine–amino acid/peptide conjugates, as well as on quinine itself, against the blood stage of the *P. falciparum* strain 3D7 (Table 3). Quinine was highly effective, having an IC_50_ value of 18 nM. The highest IC_50_ conjugates were acylbenzotriazoles-aspartic acid-quinine (compound **10**) at 17 nM and acylbenzotriazoles-L-isoleucine-glycine-quinine (compound **14**) at 23 nM (Figure 6), which have a comparable antimalarial activity to that of quinine. These findings suggest that the conjugation of short peptides with the hydroxyl group of quinine does not affect its antimalarial properties [51].

### 2.2. Flavonoids

Flavonoids are found in vegetables and fruits and have health-promoting properties, without causing substantial adverse effects [53,54]. Flavonoids have been discovered to have a wide range of biological properties, including antioxidant, antibacterial, antimalarial, antiviral and anticancer properties [55,56,57]. A typical phenylchromen-4-one scaffold is generally found in flavonoids. [58].

Despite the fact that flavonoids have positive health effects, the therapeutic results still depend on the quality of these substances’ pharmacokinetic profiles following administration. Even though they are in the form of glycosides, flavonoids are poorly bioavailable, with a low water solubility, and are rapidly altered by environmental elements, including temperature, pH and light. The methods by which flavonoids are absorbed via the gastrointestinal tract are intricate, and they are not well-absorbed in the intestine in their native state [59].

Therefore, the modification of their structures is a potentially useful option that can be used to improve their pharmacokinetic profile and also construct derivatives that are more biologically potent than their predecessor compounds. Herein, the actuality of the modifications of quercetin, curcumin and icaritin is described. The novel compounds’ preparation involves the formation of linkages between these flavonoids and specific amino acids.

#### 2.2.1. Quercetin

**Figure 7:**
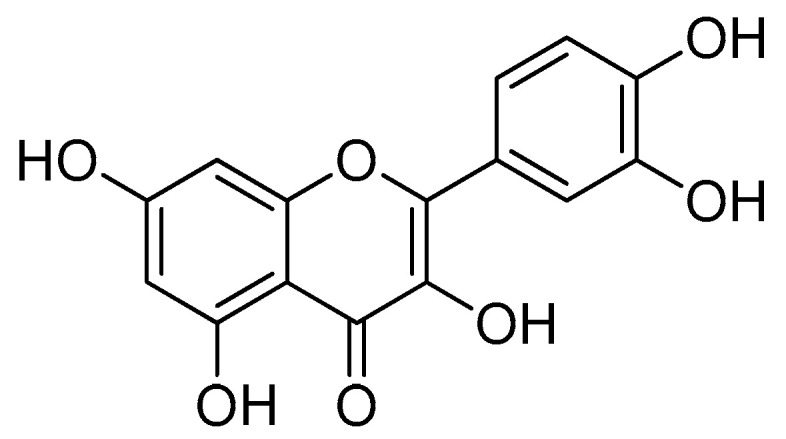
Quercetin.

Quercetin (Figure 7) is one of the compounds from natural ingredients that shows several effective biological activities [60,61]. Quercetin belongs to the flavanol group and is widely found in daily drinks, such as grapes, onions, tea and so on [62,63]. The bioavailability of quercetin is quite low in the blood plasma. In addition, the pure form of quercetin is easily excreted [64,65].

One way to overcome this problem is to synthesise quercetin–amino acid conjugates in the hope that the prodrug, quercetin, will have an improved water solubility and reduced rate of hydrolysis [66]. The conjugation of amino acids with quercetin not only serves to improve its physicochemical properties but also to overcome the resistance to the use of anticancer drugs, or what is commonly called cancer multidrug resistance (MDR). The inhibition of the drug efflux by Pgp and Pgp ATPase assays showed that amino acid–quercetin conjugates interact with the drug-binding site of Pgp to stimulate its ATPase activity [67].

**Scheme 5:**
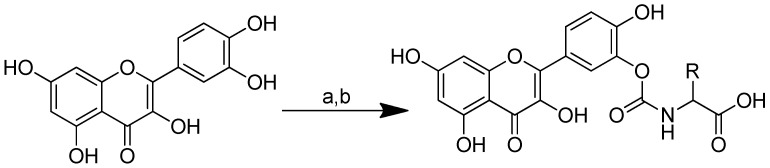
Syntheses of the quercetin analogues. (**a**): (4-NO_2_-PhO)_2_CO, DIPEA, DMF, 0 °C to rt; (**b**): TFA, CH_2_Cl_2_, 0 °C, rt. R in order: (**2**) L-alanine; (**3**) L-valine; (**4**) L-lysine; (**5**) L-phenylalanine; (**6**) L-aspartic acid; (**7**) L-methionine; (**8**) L-glutamic acid; (**9**) L-alanine-L-aspartic acid; (**10**) L-alanine-L-glutamic acid.

Quercetin–amino acid conjugates are produced through the synthesis of quercetin with nonpolar amino acids (alanine, valine, phenylalanine and methionine), which are positively charged (lysine) and negatively charged (aspartic acid, glutamine) by binding the amino acid to the B-ring of quercetin via a carbamate linker. Due to the acidity, high oxidability and reactivity of the catechol moiety, the protected amino acids’ interaction with excess quercetin primarily caused the esterification of the B-ring hydroxyls [68]. The series of quercetin–amino acid conjugates were produced as follows: synthetic amino acid carbamate derivatives of quercetin were produced through the conversion of amino acids to the corresponding activated urethanes, followed by alcoholysis with quercetin. At room temperature, amino acids, bis(4-nitrophenyl) carbonate and *N, N*-diisopropylethylamine were added to the solution. The mixture was agitated for a further 12 h at room temperature after quercetin was added. To produce the required quercetin prodrugs, the *tert*-butyl-amino acid quercetin carbamates were deprotected with TFA at 0 °C (Scheme 5) [66].

The water solubilities of the quercetin prodrugs increased dramatically (6.8–53.0 times) in comparison to quercetin (Table 4). Amino acids such as aspartic acid, glutamic acid and alanine-glutamic acid enhanced the water solubilities of their corresponding quercetin conjugates by 45.2-, 53.0- and 52.6-fold, respectively.

**Figure 8:**
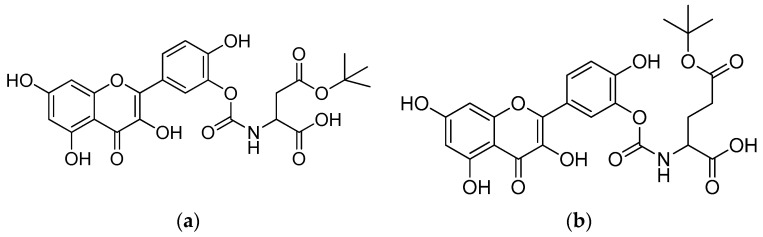
(**a**) Quercetin–aspartic acid conjugate; (**b**) quercetin–glutamic acid conjugate.

In PBS buffer (t1/2 > 17 h), the quercetin prodrugs were stable, albeit that they were vulnerable to enzymatic hydrolysis in cell lysate containing different activated hydrolysing enzymes. The quercetin–glutamic acid conjugate (Figure 8b) demonstrated a remarkable resistance to hydrolases, resulting in a much longer half-life (180 min). In comparison to quercetin, the quercetin–aspartic acid and quercetin–glutamic acid conjugates showed an increased intestinal permeability in MDCK cells, which suggests that the quercetin–amino acid conjugates may be recognised and transported by the human peptide transporter hPepT1 [66].

#### 2.2.2. Icaritin

**Figure 9:**
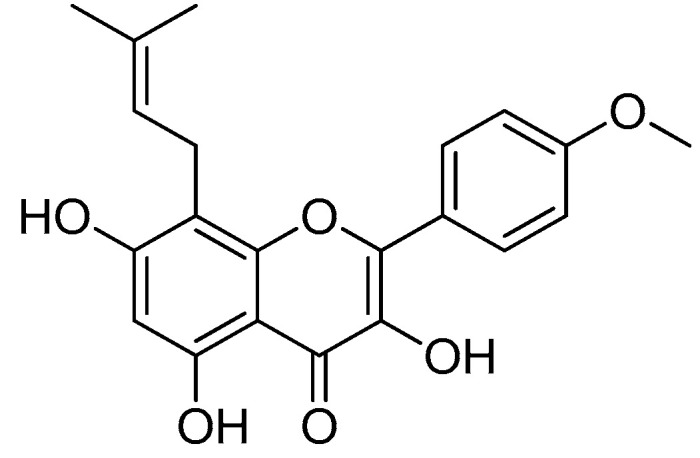
Icaritin.

Icaritin (Figure 9), an active prenyl flavone derived from *Epimedium* plants, has a wide range of pharmacological and biological properties, including those that can aid in cancer and osteoporosis treatments [69,70]. 

**Scheme 6:**
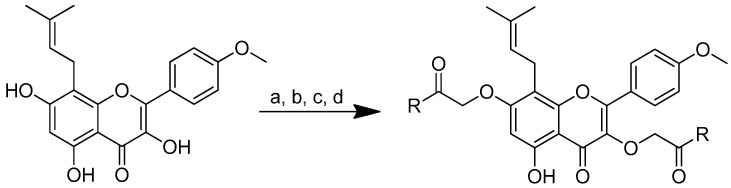
Synthesis of amino-acid-conjugated flavone compounds. (**a**) Ethyl iodoacetate, K_2_CO_3_, acetone, reflux, 12 h; (**b**) LiOH, THF, H_2_O, RT, 1.5 h; (**c**) corresponding basic amino acid, DIC, HOBt, anhydrous DMF, RT, overnight; (**d**) corresponding basic amino acid, HATU, DIPEA, anhydrous DMF, RT, overnight. R in order: (**2**) L-histidine; (**3**) L-arginine; (**4**) L-lysine.

As icaritin has a low toxicity and a high safety profile, it is a good natural chemical entity for further modification, aiming to produce molecules with an improved activity. The icaritin flavone scaffold was first alkylated with ethyl iodoacetate to integrate cationic moieties, which led to the production of flavone amphiphiles and robust bacterial membrane disruption. The key acid was then linked to the appropriate basic amino acids using 2-(3H-[1–3]triazolo [4,5-b]pyridin-3-yl)-1,1,3,3-tetramethylisouronium hexafluorophosphate (HATU) or *N,N’*-diisopropylcarbodiimide (DIC) in combination with HOBt to form icaritin–amino acid conjugates (Scheme 6) [71,72].

Cationic moieties should have an impact on the cLogP values of icaritin–amino acid conjugates. The hydrophobicity decreases as the total charge increases. Icaritin–arginine’s cLogP value is 1.65. These findings show that this conjugate’s charge–hydrophobicity balance is a key determinant of its antibacterial activity. If the total charge is too high or too low, the chemical will disrupt the charge–hydrophobicity balance, in addition to decreasing the affinity for the bacterial membranes and overall antimicrobial activity. Higher overall charges improve the antibacterial activity.

**Figure 10:**
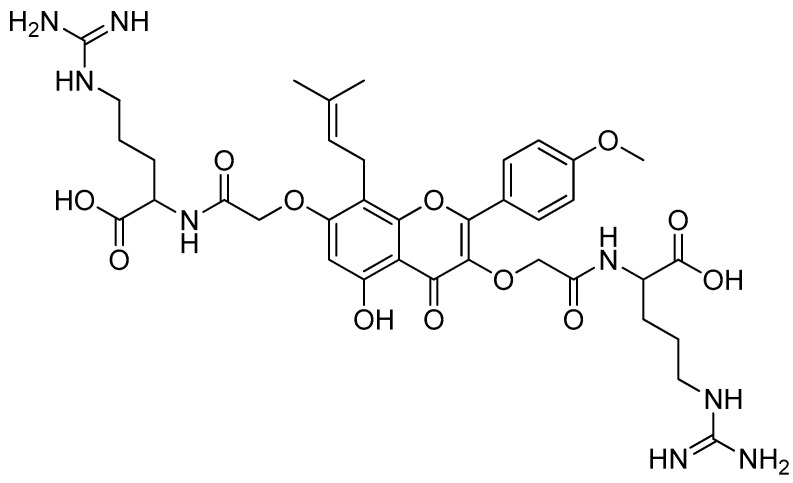
Icaritin–arginine conjugate.

Of all the substances examined, the icaritin–arginine (Figure 10) conjugate was the most effective. Icaritin–arginine was the only studied flavone derivative that was water-soluble. The strong electrostatic contact between icaritin–arginine and the negatively charged bacterial membrane was thought to be responsible for the greatest enhancement in its antibacterial activity. The guanidinium group on arginine has a more evenly distributed positive charge than the tertiary amine and -NH_2_ groups, which significantly improves icaritin–arginine’s electrostatic contact with the bacterial membrane.

Further testing of the icaritin–arginine conjugate was performed on Gram-positive bacterial strains (Table 5). The MICs ranged from 1.5 to 3.13 g/mL, which are comparable with vancomycin’s MICs of 0.78 to 1.56 g/mL. Additionally, icaritin–arginine demonstrated a remarkable efficacy against drug-resistant bacteria, indicating that icaritin–arginine does not exhibit cross-resistance with other antibiotic classes and highlighting the effectiveness of icaritin–arginine as a membrane-targeting antibiotic [72].

By damaging the bacterial membrane’s integrity and hindering the development of bacterial resistance, the conjugate killed the bacteria quickly, with an impressive membrane selectivity. In addition, icaritin–arginine showed a strong in vivo adequacy in a mouse keratitis of MRSA and *S. aureus* contaminations, subsequently showing that icaritin–arginine has the potential as an antimicrobial agent to overcome resistance problems [72].

### 2.3. Curcumin

**Figure 11:**
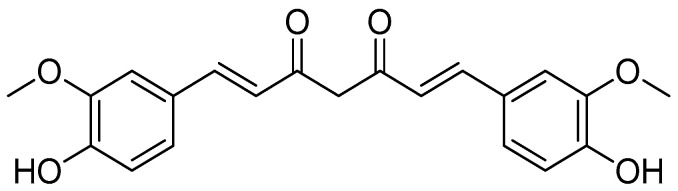
Curcumin.

Curcumin (Figure 11) and two related chemicals, i.e., demethoxycurcumin and bis-demethoxycurcumin (curcuminoids), are the primary secondary metabolites of *Curcuma longa* and other *Curcuma* species [73,74]. Curcumin, as a natural product, has several biological actions, such as anti-inflammatory, antibacterial and anticancer activities [75,76].

Clinical investigations using curcumin did not show great benefit due to its limited absorption, fast metabolism, intrinsic instability and hydrophobic nature [77,78]. Curcumin was conjugated with amino acid to enhance its intracellular accumulation, sustain its antioxidant activity and produce novel conjugates that could be more effective against biological targets [3,79,80].

**Scheme 7:**
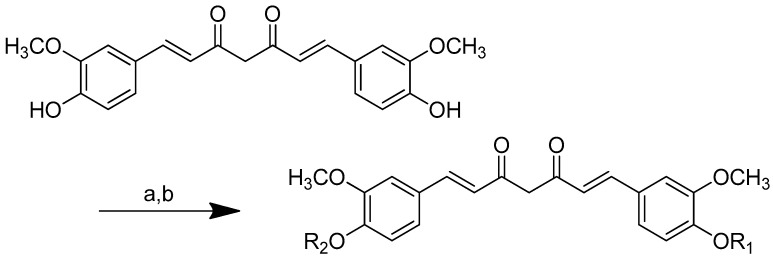
Synthesis of (**2**) 4,40-(di-O-glutamoyl)-curcumin, (**3**) 4,40-(di-O-valinoyl) curcumin, and (**4**) 4,40-(di-O-glycinoyl) curcumin. (**a**): Pyridine/N-Phathloyl (glutamoyl/valinoyl/glycinoyl) chloride/6 h/r.t.; (**b**): NH_3_:Pyridine (9:1 *v/v*).

**Scheme 8:**
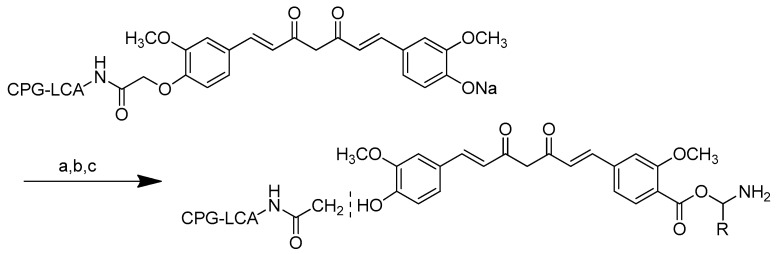
Synthesis of monoester curcumin on CPG-LCAA. (**a**): i. N-Phthaloyl ((**5**) glycinoyl/(**6**) valinoyl) chloride, DMAP, overnight, rt; (**b**): ammonia:pyridine (9:1 *v/v*), 5 min; (**c**): i. HI, 37%, 2 mL.

Several different methods were applied in order to produce curcumin conjugates, such as the formation of an *N*-phthaloyl group as a protective group (Scheme 7) [78], the binding of one of the free hydroxy groups to an insoluble polymer resin (CPG-LCAA) (Scheme 8) [78] and the use of protected amino acids, i.e., either Boc [79,80], Cbz or Fmoc [3].

Different curcumin conjugates were produced, including 4,40-(di-*O*-glutamoyl) curcumin, 4,40-(di-*O*-valinoyl) curcumin, 4,40-(di-*O*-glycinoyl) curcumin, monoglycinoyl curcumin and monovalinoyl curcumin. Diester curcumin was synthesised using a synthetic approach, as shown in Scheme 7. The NH_2_ group of amino acids (glycine, valine, and glutamic acid) were protected as *N*-phthaloyl derivatives during diester synthesis. To obtain the corresponding acid chlorides, the carboxyl group was activated by treating it with thionyl chloride. Curcumin was suspended in dry pyridine and treated with the *N*-phthaloyl chlorides of the respective amino acids in a 1:2.5 molar ratio for six hours. Ammonia was used to remove the phthaloyl group, and column chromatography was utilised to purify the conjugates [78].

The curcumin monoesters were synthesised by anchoring one of the free phenolic groups to an insoluble polymeric solid support resin long-chain alkylamine controlled pore glass (LCAA-CPG) via a two-carbon linker in the solid phase (Scheme 8). One of curcumin’s phenolic groups was linked to LCAA-CPG via chloroacetic acid as a linker, while the carboxy functional group of chloroacetic acid was activated with *p*-nitrophenol in the presence of pyridine and triethylamine (basic environment) to obtain the corresponding activated ester. The amide bond was formed by reacting the activated ester of chloroacetic acid with the amino function of LCAA-CPG using DCC/DMAP. A symmetrical sodium salt of curcumin was added to the reaction with chloroacetic-acid-derivatised LCAA-CPG, forming a bond with one phenolic group while the other remained free for further synthesis. On a solid support, the *N*-phthaloyl chlorides of glycine and valine were reacted with the sodium salt of curcumin to produce the corresponding curcumin monoesters. Ammonia was used to remove the phthaloyl group. By cleaving the ethereal bond, HI was used to deblock the monoester from the solid support, allowing for the selective esterification of curcumin with glycine and valine (Scheme 8).

**Figure 12:**
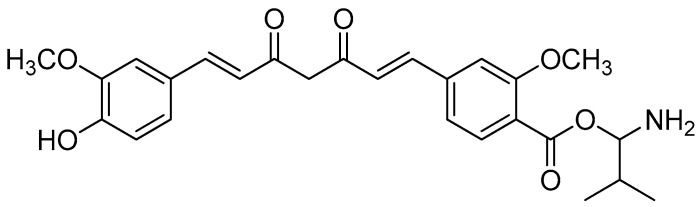
Monovalinoyl curcumin.

The curcumin bioconjugates, including diesters and monoesters, were tested for their antibacterial activity against multi-resistant bacteria that cause secondary infections in humans, such as *E. cloacae*, *S. saprophyticus*, *Micrococci*, *K. aeruginosa* and *E. coli*. The inhibition zone findings of the assay showed promising results. A disk containing 20 mmol of Amoxyclav^®^ was used, and the conjugates were loaded onto separate disks in equal amounts. Amoxyclav^®^ showed a 20 mm zone of inhibition, whereas monovalinoyl curcumin (Figure 12) had an inhibition zone of 26 mm (Table 6). Monovalinoyl curcumin showed the most promising findings, with MICs of 2.5 mmol/mL against *Micrococcus* and *E. cloacae*, while Amoxyclav^®^, which is the most widely used antibiotic, had an MIC of 10 mmol/mL.

These findings imply that curcumin diesters have a greater antibacterial activity than curcumin itself, which could be related to their higher solubility, improved cellular uptake (bioavailability) and slower metabolic processes due to the masking of the free phenolic groups. Monoesters, on the other hand, have a higher activity than their corresponding diesters because they have both advantages, namely, a ligand to aid in cellular uptake and a free phenolic group for binding at the active site [78].

**Scheme 9:**
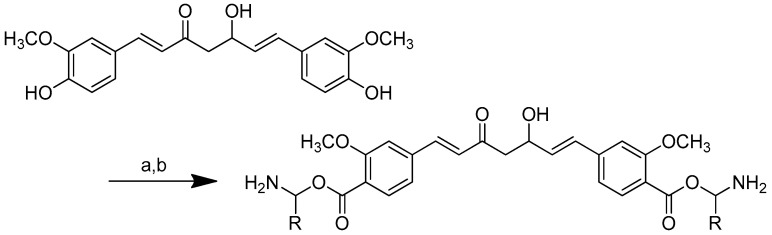
Synthesis of curcumin–amino acid conjugates. (**a**) DCC/DMAP/TEA, dioxane, N_2_, 25–30 °C; (**b**) TFA (10% solution, DCM), 10 min.

The *t*-Boc moiety was designed to protect the carboxyl group in amino acids during their reaction with curcumin (Scheme 9). Curcumin was reacted with *t*-Boc-protected amino acids in dry dioxane in the presence of dehydrating agents, such as *N, N’*-dicyclohexylcarbodiimide (DCC), 4-dimethylamino-pyridine (DMAP) and triethylamine (TEA). Because higher reaction temperatures resulted in very low product yields, the reaction was carried out in a nitrogen atmosphere at ambient temperatures, such as 25–30 °C, combined with stirring until the reaction was completed (8–12 h).

The product in the organic layer was isolated after the reaction mixture was filtered in order to remove dicyclohexylurea (DCU) as a by-product. TFA was used to deprotect the *t*-Boc amino acid–curcumin conjugates in dry dichloromethane under ultrasonic conditions for 10 min. The method was gentler than the standard protocol for *t*-Boc deprotection, which used TFA with a reaction time of 1–2 h. The resulting compounds were obtained in a pure form, with an overall yield of 45–76%.

**Figure 13:**
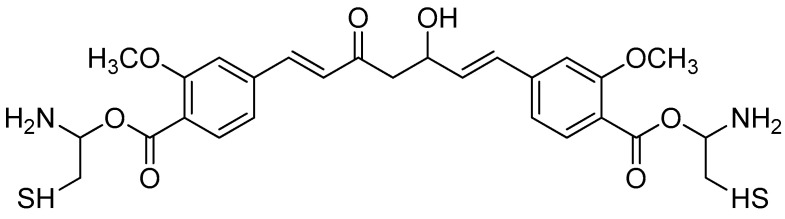
Biscysteinoyl curcumin.

Curcumin conjugates contain alkyl-substituted amino acids (Scheme 9). In particular, the cysteine conjugate (Figure 13) showed a significantly higher antioxidant activity than curcumin. The DPPH radical-scavenging assay clearly showed that the curcumin–amino acid conjugate had lower IC_50_ values than curcumin. The curcumin–cysteine conjugate had 70% lower IC_50_ values than curcumin. The antioxidant activity, as measured by beta-carotene bleaching assays, revealed that the cysteine derivative had lower IC_50_ values than the parent molecule. Based on these results, the cysteine conjugate demonstrated a high activity, which also confirms the role of the sulphur moiety in free radical scavenging. As both methods yielded similar results, this shows that the curcumin–cysteine conjugate, indeed, is more potent than unconjugated curcumin [79].

**Scheme 10:**
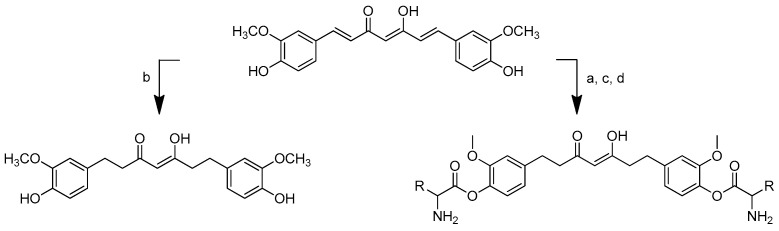
Synthesis of tetrahydrocurcumin–amino acid conjugates. (**a**) DCC, DMAP, dioxane, rt, 7–12 h, inert; (**b**) Pd/BaSO4; (**c**) 10% TFA in DCM, 0 °C, 3–5.5 h; (**d**) H2, Pd/BaSO4, 30 psi, 0.5–1.5 h. R in order: (**2**) L-alanine; (**3**) L-isoleucine; (**4**) L-proline; (**5**) L-valine; (**6**) L-phenylalanine; (**7**) L-glycine; (**8**) L-leucine.

Tetrahydrocurcumin (THC)–amino acid conjugates also showed a significantly better antibacterial activity than tetrahydrocurcumin against all the microorganisms tested. The synthesis of the amino acid–tetrahydrocurcumin derivatives was performed via two routes (Scheme 10). The first involved the conversion of curcumin to tetrahydrocurcumin using the reductor Pd/BaSO_4_. The mixture was churned under 30 psi H_2_ pressure until the completion of the reaction (0.5–1.5 h). Meanwhile, in the other pathway, curcumin was conjugated firstly with Boc-protected amino acid followed by the gradual addition of 4-dimethylaminopyridine (DMAP) and *N, N’*-dicyclohexylcarbodiimide (DCC) in dioxane. The mixture was stirred for 7–12 h under nitrogen. The curcumin–amino acid *t*-Boc conjugates were deprotected by TFA in CH_2_Cl_2_ under ice-cold conditions for 3–5.5 h. The conjugates were then transformed into tetrahydrocurcumin–amino acid conjugates through the addition of Pd/BaSO_4_ under 30 psi H_2_ pressure for 0.5–1.5 h [80].

The activity of THC and its amino acid conjugates towards two Gram-positive (*B. cereus* and *S. aureus*) and two Gram-negative bacteria (*E. coli* and *Y. enterocolitica*) was investigated (Table 7).

**Figure 14:**
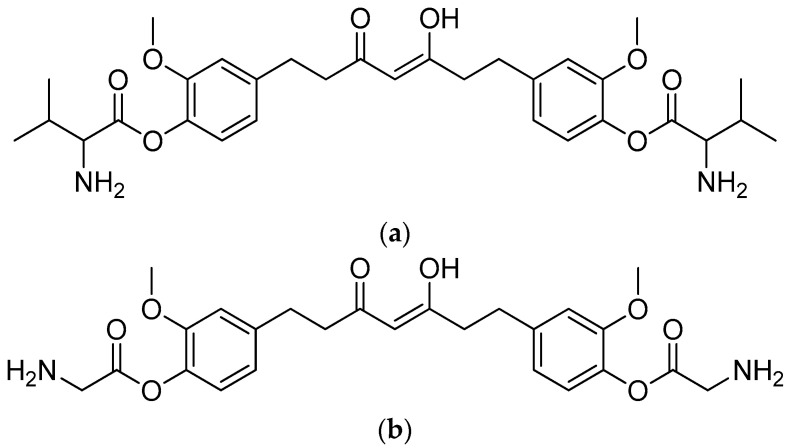
(**a**) Tetrahydrocurcumin–L-glycine; (**b**) tetrahydrocurcumin–L-valine.

All of the substances had noticeably greater actions than THC. Due to their low MIC values of 257 and 263 µM, respectively, against *B. cereus*, tetrahydrocurcumin–glycine and tetrahydrocurcumin–valine were reported to be the most powerful conjugates among the seven conjugates produced (Figure 14).

Prior research indicated that THC–amino acid conjugates have a significantly stronger antibacterial activity than THC against all of the examined microorganisms [80]. The amino acid component of the derivatives apparently made the conjugates more hydrophilic and aided in the cellular uptake of the covalently bound THC by microorganisms. Thus, their greater concentration in the bacterial cells could be the reason for their greater antibacterial activity.

### 2.4. Terpenoids

The terpenoid structure consists of many isoprene units. They can be divided into monoterpene, sesquiterpene, diterpene, triterpene, tetraterpene and polyterpene based on the number of isoprene units [81]. The terpenes utilised in therapeutic applications have some pharmacological activities, such as antimalarial, anticancer and anti-infective properties. Natural terpenoids offer some intriguing qualities, but they must still be refined in order to have the potency, selectivity and pharmacokinetic characteristics of a clinically relevant medication [82,83]. The creation of semisynthetic derivatives can provide a feasible approach to optimising the base scaffold to enhance their activities.

#### 2.4.1. Astaxanthin

**Figure 15:**
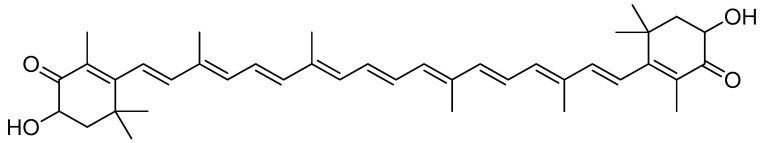
Astaxanthin.

Astaxanthin (Figure 15) is a red fat-soluble xanthophyll carotenoid pigment that does not possess pro-vitamin A activity in humans. However, some investigations showed that it has an enhanced biological action compared to other carotenoids [84,85]. Astaxanthin is present in several living creatures, including salmon, trout, crayfish and prawns, many of which are found in the maritime ecosystem [86,87]. Fungi such as *Phaffia rhodozyma* and *Xanthophyllomyces dendrorhous* are also among the sources of astaxanthin [88,89].

The modification of the chemical structure of the esterified moieties can improve the water solubility and/or dispersibility of synthetic carotenoid analogues [90]. A highly water-dispersible astaxanthin derivative was produced by means of the esterification of the carboxyl group of amino acid lysine, and then the product was converted to the tetrahydrochloride salt [91].

**Scheme 11:**
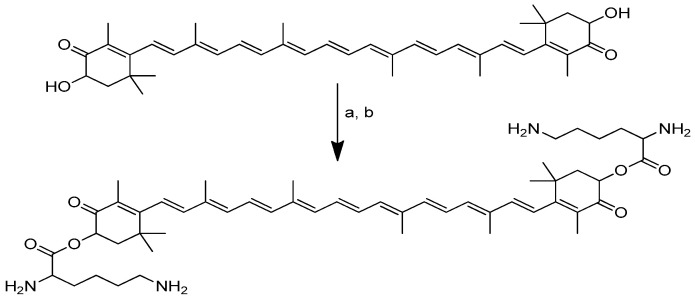
Synthesis of the dilysinate diester of astaxanthin. (**a**) Boc-protected lysine, DMAP, DIC, CH_2_CL_2_; (**b**) anhydrous HCl in dioxane.

The conjugation of dilysine with astaxanthin via ester linkage is accomplished in two steps. The first step is carried out through the reaction of Boc-protected lysine with astaxanthin using carbodiimide as a catalyst (DMAP and DIC). Deprotection is carried out in the next step using anhydrous hydrochloric acid in dioxane (Scheme 11) [91].

The tetrahydrochloride salt of dilysinate astaxanthin was synthesised in two steps, yielding a molecule with improved value as an aqueous phase and/or in vivo therapeutic antioxidant and radical scavenger. No heat, detergents, co-solvents or other additions were used to achieve aqueous dispersibility (>181.6 mg/mL). This constitutes the most significant improvement in the aqueous dispersibility. Direct superoxide scavenging was shown to be effective, with the near-complete suppression of the superoxide signal obtained at a concentration of 100 µM. This molecule may be useful in biological and chemical applications that require aqueous phase radical scavenging [91].

#### 2.4.2. Oleanolic Acid

**Figure 16:**
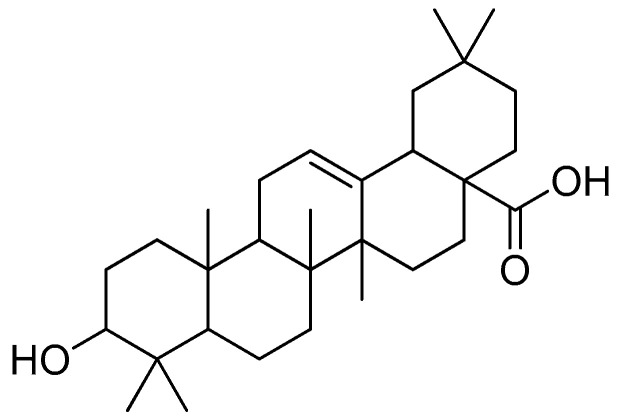
Oleanolic acid.

Oleanolic acid (Figure 16) is a pentacyclic triterpenoid that can be found in a variety of foods and medicinal plants [92,93]. Oleanolic acid is widely known for its hepatoprotective properties in the treatment of acute chemical-induced liver injury, chronic liver fibrosis and cirrhosis [94,95,96]. Oleanolic acid showed biological activity against H1N1 virus infection in MDCK cells by disrupting the link between the viral protein and its receptors, preventing the virus’ attachment to the host cells [97].

To improve the hydrophilicity, alkalinity and biological activity of oleanolic acid, a variety of novel oleanolic acid–amino acid conjugates were synthesised using the ester condensation technique to incorporate different amino acids into the 3-hydroxyl of oleanolic acid [96]. These conjugates were produced to investigate their activity against HCCs and breast cancer cell lines [98].

**Scheme 12:**
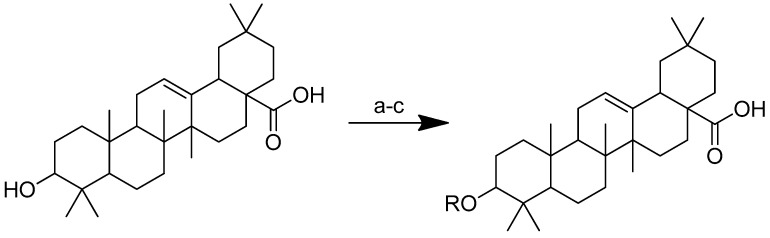
Synthesis of oleanolic conjugates. (**a**) Benzyl bromide, K_2_CO_3_, DMF, reflux 2 h, 85 °C; (**b**) Cbz-(L-)-amide acids, EDCI, DMAP, dry DCM, r.t. 12 h; (**c**) Pd(OH)_2_/C, H_2_, MeOH, r.t. 12 h.

Scheme 12 depicts the synthesis of oleanolic acid conjugates. Cbz-protected amino acid and oleanolic acid were used as the materials to create the conjugates. In DCM, EDCI/DMAP was used to facilitate the process of ester condensation. The protective group was then deprotected using Pd(OH)_2_/C at the same time [96].

**Figure 17:**
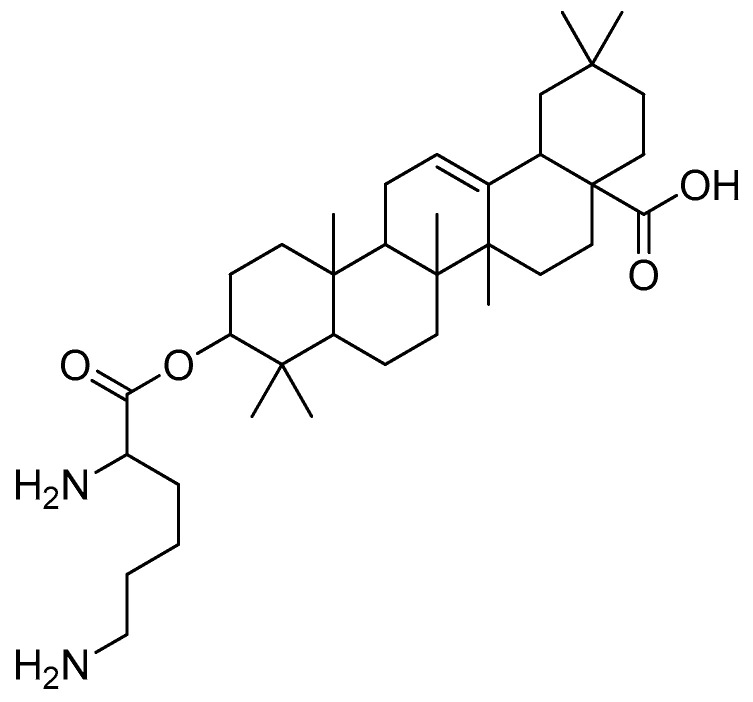
Oleanolic acid–lysine conjugate.

After introducing various amino acids to the 3-hydroxyl of oleanolic acid, the ClogP of the derivatives was lowered to a considerable extent, with the glycine, alanine and lysine conjugates showing the most significant effects. The oleanolic acid–lysine conjugates (Figure 17) showed a dramatic increase in apoptosis via the mitochondrial apoptotic pathway and successfully lowered the enzymatic activity of ALT and AST in the serum. They also demonstrated hepatoprotective effects on the CCl_4_-induced acute liver damage mouse model.

Furthermore, the oleanolic acid–lysine conjugate also showed better biological properties than oleanolic acid (20 mg/kg, intragastric injection) in the in vivo model. These findings suggest that basic amino acids (lysine) could effectively increase oleanolic acid’s hydrophilicity and alkalinity, in addition to altering the extracellular weak acidic microenvironment that, in turn, improves its overall bioavailability.

#### 2.4.3. Betulin

There are two pentacyclic triterpene natural products, known as betulin and betulinic acid, that were reported as secondary metabolites in several plant species [99]. Betulin, betulinic acid and their by-products have a wide range of pharmacological effects, including anticancer, anti-inflammatory and antiparasitic properties [100]. The more bioactive structure is that of betulinic acid, while betulin is also widely available and fairly simple to separate from birch tree bark. Unfortunately, because both betulinic acid and its metabolic precursor, i.e., betulin, are extremely poorly soluble in aqueous buffers, their bioavailability and biodistribution are unsuitable for use in medicine [101].

**Scheme 13:**
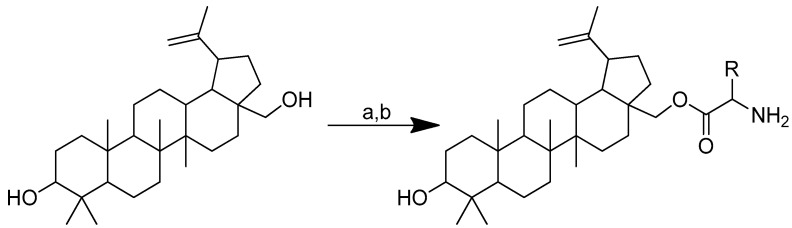
The synthesis of monosubstituted betulin esters containing L-amino acids. (**a**) Boc-amino acids, CDI, THF, 24 h; (**b**) HCl in methanol. R in order: L-lysine; L-ornithine.

A study revealed that betulin may be more water-soluble when it is conjugated with specific natural amino acids. In addition, the conjugate also exhibited proapoptotic activity in cancer cells. Boc-protected L-amino acid was used to create amino acid esters of betulin conjugates in anhydrous THF and CDI (Scheme 13). The mixture was preincubated at room temperature for 30 min. Thereafter, betulin was added and agitated for 24 h in an environment of inert gas. To obtain pure, Boc-protected, monosubstituted betulin esters, THF was evaporated under low pressure once the reaction was complete. The Boc group was then removed in the following step using HCL in methanol [102].

A431 is a human epidermal cancer cell line that was targeted by the amino acid–betulin conjugates, with regular keratinocytes (HaCaT) as the control. A comparison between the novel betulin esters and their unaltered antecedents revealed an improved anticancer activity (Table 8).

**Figure 18:**
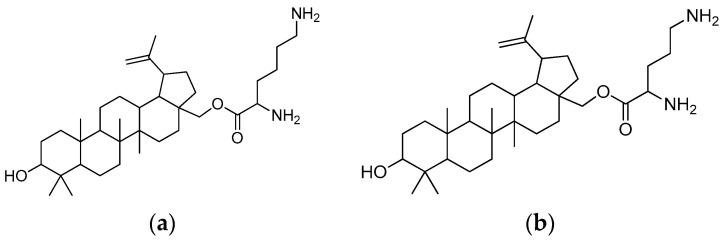
(**a**) Betulin-L-lysine; (**b**) betulin-L-ornithine.

In a comparison with betulin and betulinic acid, compounds derived with lysine and ornithine residues (Figure 18) showed improved cytotoxicity in A431 cancer cell lines (IC_50_ 2.3 μM and 4.5 μM after 72 h, respectively). The opposite reaction was observed in the HaCaT control cells. The most advantageous aspect of the novel compounds is that they are harmless to typical human keratinocytes, and it is important to emphasise their specific toxicity towards epidermoid carcinoma cells.

#### 2.4.4. Glycyrrhetinic Acid

**Figure 19:**
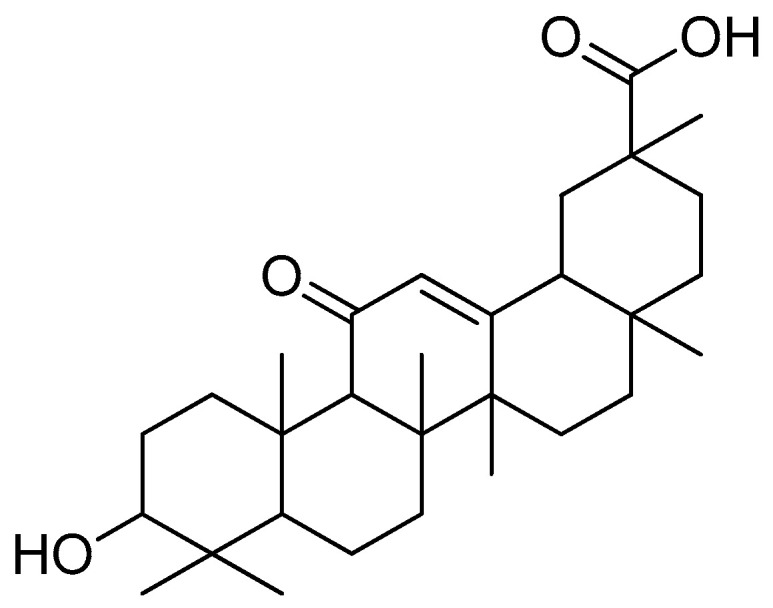
Glycyrrhetinic acid.

The natural substance glycyrrhetinic acid (Figure 19), which is prevalent in liquorice root, has a triterpenoid aglycone component called glycyrrhetinic acid [103]. Research has shown that this compound has a wide range of outstanding biological activities, including anti-inflammatory, antiviral, hepatoprotective and anticancer characteristics [104,105].

Compared with other triterpenes, such as triptolide and betulinic acid, the anticancer activity of glycyrrhetinic acid can be classified as moderately active. Modifications of its structure may lead to the discovery of new derivates that might be more effective as antitumor agents [106,107].

**Scheme 14:**
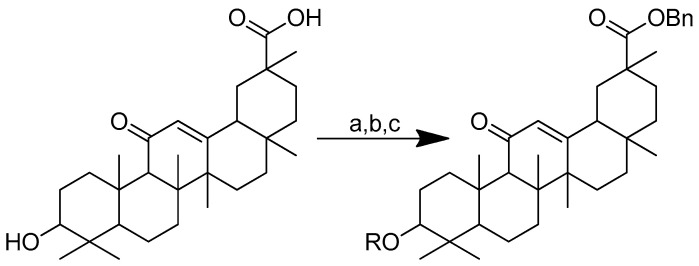
Synthesis of the glycyrrhetinic acid derivatives with esters at the C-3 position: (**a**) Bn-Br, dry DMF, dry K_2_CO_3_, 85 °C, reflux, 3 h; (**b**) Boc-amino acids, DCM, DMAP, EDCI, rt, 12 h; (**c**) TFA in dry DCM. R in order: (**3**) L-glycine; (**4**) L-alanine; (**5**) L-phenylalanine; (**6**) L-proline; (**7**) L-sarine; (**8**) L-leucine; (**9**) L-isoleucine; (**10**) L-methionine.

Glycyrrhetinic acid–amino acid derivatives have reportedly been synthesised to increase the cytotoxicity of the acid. The modification was conducted through an alteration at C-30 by esterification with benzyl ester (Scheme 14). Two distinct series of derivatives were successfully created. Glycyrrhetinic acid with esters in the C-3 position and glycyrrhetinic acid with amide linkages in the C-3 position are the two aforementioned categories of derivatives. The two pathways used to manufacture the derivatives are ester linkage-based and amide linkage-based. The first pathway involves the esterification of glycyrrhetinic acid at the C-3 position in the presence of benzyl bromide and K_2_CO_3_ at 85 °C in *N, N*-dimethylformamide to produce an intermediate, i.e., glycyrrhetinic acid-benzyl ester. The intermediate was then subjected to further reactions with N-protected-L-amino acid in dry dichloromethane (DCM) in the presence of 1-ethyl-3-(3-dimethylaminopropyl) carbodiimide hydrochloride (EDCI) and 4-dimethylaminopyridine (DMAP). Afterwards, trifluoroacetic acid (TFA) deprotection was carried out in dry DCM at 0 °C.

**Scheme 15:**
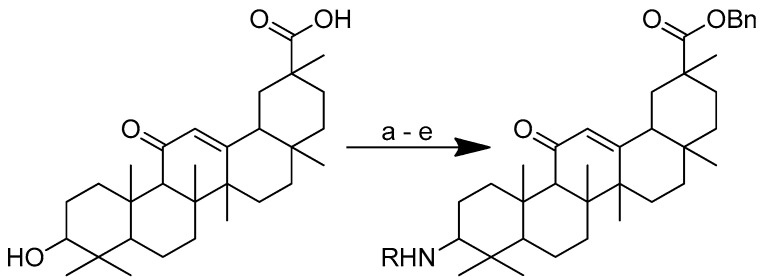
Synthesis of the glycyrrhetinic acid with amide linkages at the C-3 position. Reagents and conditions: (**a**) Bn-Br, dry DMF, dry K_2_CO_3_, 80 °C, reflux, 3 h; (**b**) CrO_3_/H_2_SO_4_, CH_3_COCH_3_, 0 °C, 1 h; (**c**) NaCNBH_3_, CH_3_COONH_4_, CH_3_OH, rt, 12 h; (**d**) Boc-amino acids, DCM, HOBt, EDCI, DIPEA, rt, 12 h; (**e**) TFA in dry DCM, 0 °C, 4 h. R in order: (**3**) L-glycine; (**4**) L-alanine; (**5**) L-phenylalanine; (**6**) L-proline; (**7**) L-sarine; (**8**) L-leucine; (**9**) L-isoleucine; (**10**) L-methionine.

In the second pathway, the derivatives were prepared through amide bond formation at the C-3 position (Scheme 15). In the first step, the carboxylic acid was protected before being oxidised in acetone with CrO_3_/H_2_SO_4_. By reducing the resulting intermediate with sodium cyanoborohydride and ammonium acetate in methanol, the glycyrrhetinic acid-benzyl ester epimer containing amide connections in the C-3 position was created, which was then coupled with N-protected L-amino acid in dry DCM under the catalysis of EDCI, 1-hydroxybenzotriazole (HOBt) and *N,N*-diisopropylethylamine (DIPEA). TFA deprotection was carried out in dry DCM at 0 °C [108]. All the substances were tested for their cytotoxic potential against human A549 cancer cell lines (lung cancer) and MCF7 breast cancer cells.

The glycyrrhetinic acid–amino acid conjugates were found to be more effective than glycyrrhetinic acid against A549 tumour cell lines. All glycyrrhetinic acid-amino acid derivatives linked by esters showed better activity than the parent compound (Table 9).

The cytotoxicity detection also showed that the majority of the glycyrrhetinic acid–amino acid derivatives, particularly the amide linkage derivatives, had greater anticancer activities than cisplatin (as the standard). Glycyrrhetinic acid–alanine with an amide linkage (Table 10) was almost 18 times more effective than glycyrrhetinic acid (IC_50_ > 40 µM) in the inhibition of tumour growth in the A549 cell lines (IC_50_ 2.1 µM), as compared to cisplatin (IC_50_ 9 µM) [108].

**Figure 20:**
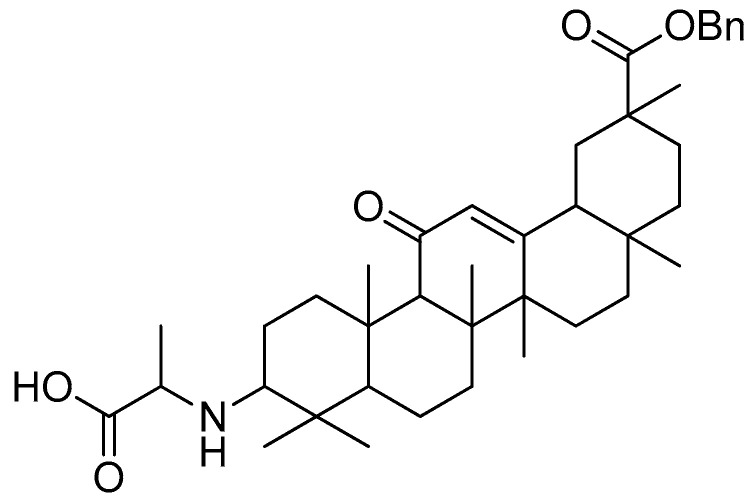
Glycyrrhetinic acid–alanine with amide linkage.

The antiproliferative effects of the amide-coupled derivatives against the tested cell lines were evidently superior to those of the ester linkage derivatives. Glycyrrhetinic acid–alanine (Figure 20) was proven to be the most effective derivative against A549 cells among all of them. As previously mentioned, converting the hydroxyl of C-3 into an amino group improved the anticancer activity of all the derivatives [108].

### 2.5. Lignans

Lignans are secondary metabolites extensively found in plants [109]. Structurally, the dimeric structures of lignan compounds are created by a β, β′ linkage between two phenylpropane units with varying levels of sidechain oxidation and distinct substitution patterns on the phenyl ring [110]. This class of compounds is known to be able to prevent a number of chronic diseases that are hormone-related, including heart disease [111], breast cancer [112] and menopausal symptoms [113].

Even though lignans have dominant pharmacological effects, they have some disadvantages, including excessive side effects, a lack of selectivity, low solubility and insufficient bioavailability. Thus, particular transformations are applied to lignan compounds to eliminate these limitations. Arctigenin and podophyllotoxin are two lignans that offer promising biological actions. Nevertheless, these two compounds still have several drawbacks. Thus, the two compounds have reportedly been conjugated with certain amino acids to improve their biological properties.

#### 2.5.1. Arctigenin

**Figure 21:**
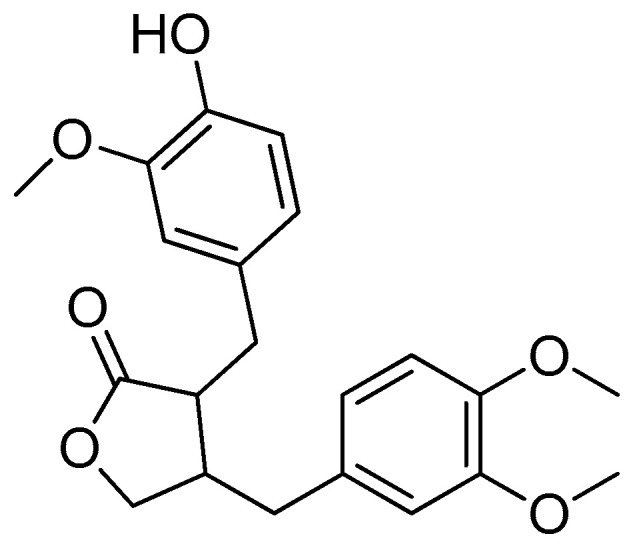
Arctigenin.

Arctigenin (Figure 21), a natural lignan found in the fruits of the *Arctium lappa* L. species, is a potentially active biomolecule [114,115]. This compound has antitumor, anti-inflammatory and antiviral properties according to the reports [116,117].

**Scheme 16:**
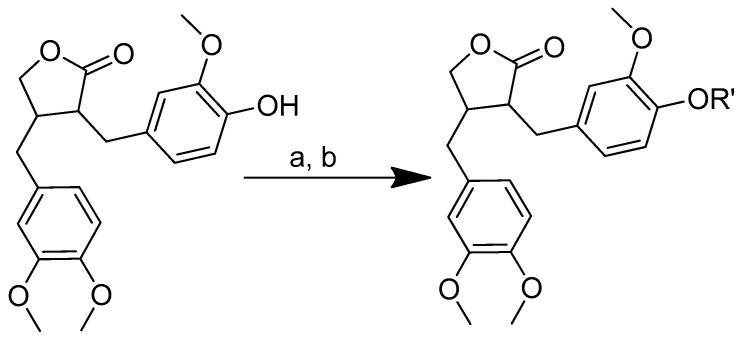
Synthesis of amino acid–arctigenin conjugates. (**a**) C_2_H_3_N, EDCI, DMAP; (**b**) CH_3_COOCH_2_CH_3_, HCl. R in order: (**3**) L-methionine; (**4**) L-phenylalanine; (**5**) L-valine.

Amino acid–arctigenin conjugates have reportedly been designed and synthesised to improve the solubility and bioavailability of arctigenin. Arctigenin–amino acid conjugates were synthesised using an amino acid–EDCI-DMAP ratio of 1:2:2:0.5 (Scheme 16). The reaction mixture was dissolved in acetonitrile and stirred for a period of one to two hours at 0 °C. The arginine–amino acid derivatives were created by deprotecting the crude products with HCl gas [118,119].

The solubility of the arctigenin–amino acid conjugates in water was assessed in accordance with the standards. The derivative compounds had a higher water solubility than arctigenin. The aqueous solubility of arctigenin was considerably enhanced following conjugation with the amino acids.

**Figure 22:**
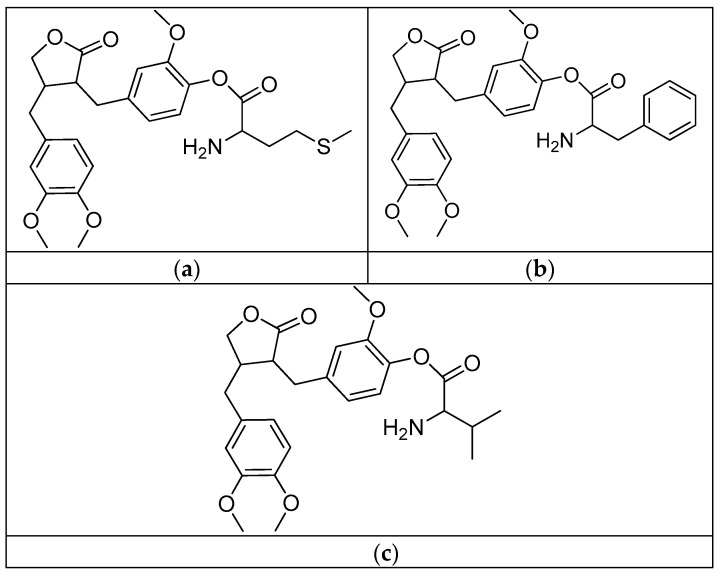
(**a**) Arctigenin-L-methionine; (**b**) arctigenin-L-phenylalanine; (**c**) arctigenin-L-valine.

These findings also showed that the conjugates of arctigenin–methionine, arctigenin–phenylalanine and arctigenin–valine have a better antitumor activity both in vitro and in vivo. All of the tumour-bearing animals in all of the groups demonstrated varied inhibitory rates. The inhibitory rates of the derivatives were higher than the inhibitory rate of arctigenin. Table 11 shows the ability of cyclophosphamide, arctigenin, arctigenin–methionine, arctigenin–phenylalanine and arctigenin–valine in suppressing the growth of mice with transplanted H22 tumours. The findings demonstrate that there is a considerable difference in tumour growth inhibition between the arctigenin group and the three experimental derivative groups in order arctigenin–methionine, arctigenin–phenylalanine and arctigenin–valine (Figure 22). Compared to cyclophosphamide (an established medication for chemotherapy), arctigenin had a significantly lesser effect on tumour suppression. Surprisingly, arctigenin–methionine, arctigenin–phenylalanine and arctigenin–valine reduced the tumour weights significantly in comparison to arctigenin. Arctigenin–valine inhibited the tumour growth equivalently to cyclophosphamide. Arctigenin–valine had a tumour-suppressing action that was almost two times better than that of arctigenin [118].

#### 2.5.2. Podophyllotoxin

An antimitotic lignan known as podophyllotoxin was discovered in the root of the podophyllum resin plant. It drew attention because of its strong biological activities, including antiviral, anthelminthic and antineoplastic properties [120,121,122]. However, its potential use was constrained by adverse consequences, such as excessive damage to healthy cells and a lack of selectivity towards diseased tissues [123,124]. Therefore, podophyllotoxin derivatives were designed and synthesised in order to enhance the antitumor and selectivity of the original compound. The cytotoxic activities of the derivatives were tested against A549 (human lung cancer), MCF-7 (human breast cancer), HepG2 (human hepatocellular carcinoma) and L-02 (human normal hepatocyte) cell lines [125].

**Scheme 17:**
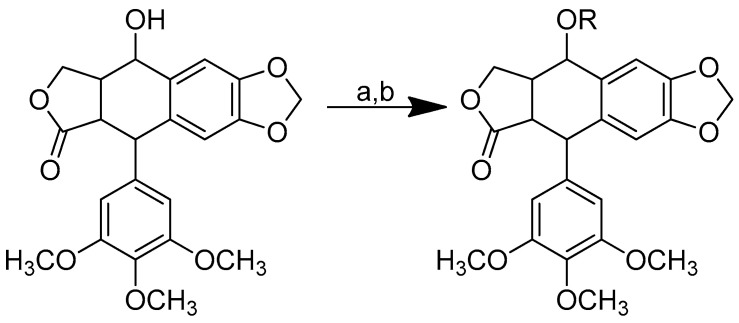
Synthesis of the podophyllotoxin–amino acid conjugates. Reagents and conditions: (**a**) Boc-amino acids, EDCI, DMAP, room temperature, 12 h; (**b**) TFA in dry DCM, 0 °C, 4 h. R in order: (**1**) Boc-L-glycine; (**2**) Boc-L-sarcosine; (**3**) Boc-L-alanine; (**4**) Boc-L-phenylalanine; (**5**) L-glycine; (**6**) L-sarcosine; (**7**) L-alanine; (**8**) L-phenylalanine.

According to Scheme 17, podophyllotoxin–amino acid conjugates were produced by conjugating podophyllotoxin with N-protected-L-amino acid using an activating agent of 1-ethyl-3-(3-dimethylaminopropyl) carbodiimide hydrochloride (EDCI) and a base catalyst of 4-dimethylaminopyridine (DMAP) in dry dichloromethane (DCM). At room temperature, the reaction was carried out for 12 h. Thereafter, trifluoroacetic acid (TFA) was used to deprotect the conjugates in dry DCM for four hours in an ice bath.

**Figure 23:**
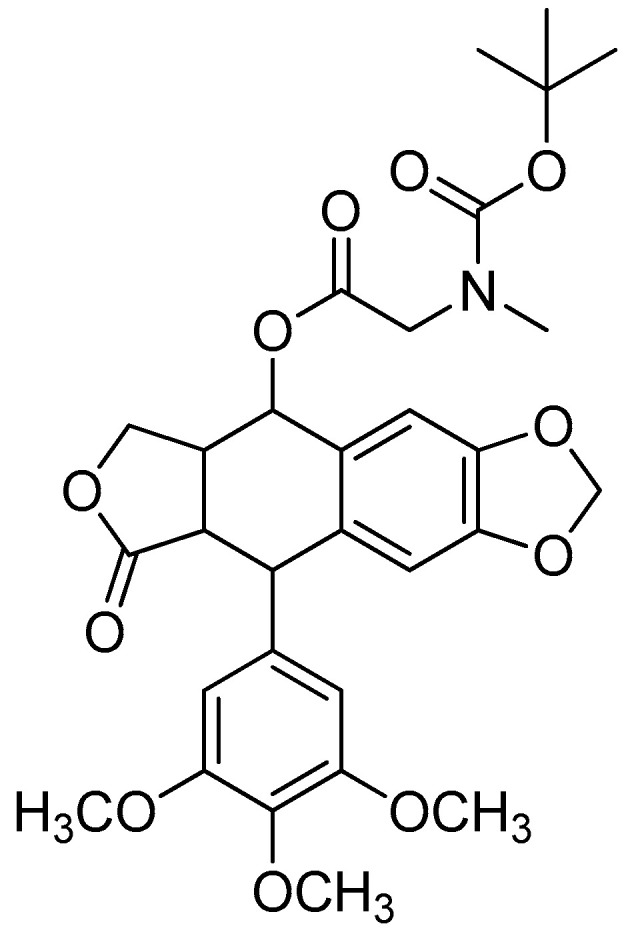
Podophyllotoxin-Boc-L-sarcosine.

The MTT assay was used to test the in vitro cytotoxicity of podophyllotoxin–amino acid conjugates on the tumour cell lines A549, MCF-7 and HepG2 and normal hepatocyte L-02. As shown in Table 12, majority of the compounds were more effective against the three tumour cell lines than doxorubicin, which is a common anticancer drug used in clinical settings. Podophyllotoxin-sarcosine-Boc (Figure 23) showed the greatest selectivity, the strongest cytotoxicity and the lowest toxicity among all of the tested compounds. According to the results of DAPI staining, this compound displayed decreased toxicity to normal hepatic L-02 cells and could cause A549 apoptosis through nuclei fragmentation [125].

### 2.6. Xanthones

Xanthone, also known as 9*H*-xanthen-9-one, with the molecular formula C_13_H_8_O_2_, is an aromatic oxygenated heterocyclic molecule with a dibenzo-γ-pirone scaffold. The word “xanthone” is derived from the Greek word “Xanthos” and refers to a yellow-coloured compound isolated from the pericarp of the mangosteen (*Garcinia mangostana* Linn.), a tropical fruit of the Guttiferae family (yellow) [126]. The structures of oxygenated heterocyclic derivatives and γ-pyrone natural compounds such as the flavonoids and chromones have strong similarities. Their antioxidant and anti-inflammatory activities have been the subject of numerous reports in the past [127].

Given its high binding affinity to numerous unrelated kinds of protein receptors, the xanthone nucleus may be regarded as a “privileged structure”. The presence of a heteroaromatic tricyclic ring system that is primarily planar and rigid, a carbonyl group at the central ring that is capable of several interactions and a biaryl ether group contributing to the electronic system, the xanthone core can accommodate a wide range of derivatisations in various positions. The derivatisation of xanthone is related to the ability of xanthones to interfere with various biological targets [128].

Xanthone has the potential to be utilised as a parent compound for the creation of novel medicinal molecules. To develop, comprehend and discover novel compounds that are effective against specific biological targets, the fundamental framework is employed as a template. The production of novel compounds based on the conjugation of various types of amino acids with the xanthone base skeleton has been the subject of numerous studies [129].

#### α-Mangostin

**Figure 24:**
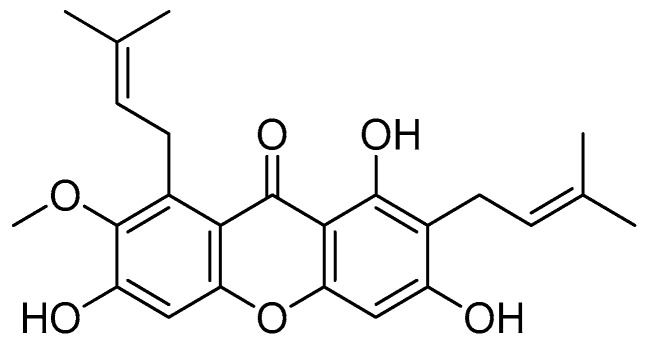
α-Mangostin.

α-Mangostin (Figure 24) is the most prevalent xanthone found in mangosteen pericarps [130]. α-Mangostin has been shown to have antioxidant, anti-infective, anticarcinogenic and antidiabetic activities, as well as neuroprotective, hepatoprotective and cardioprotective qualities, with the anticarcinogenic action being the most promising [131,132,133].

To further understand the function of cationic amino acid in the antimicrobial activity of xanthone-based derivatives, Li et al. reported the design of membrane-targeting compounds with cationic amino acids. This was based on their previous finding that shows the ability of cationic amino acids to enhance membrane selectivity [71,134].

**Scheme 18:**
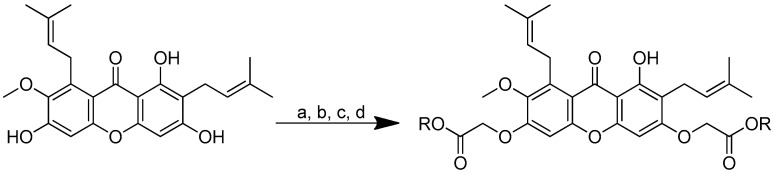
Synthetic pathway for α-mangostin-conjugated amino acids. (**a**) BrCH_2_CO_2_Me, KOH, anhydrous EtOH, reflux, 24 h; (**b**) LiOH, THF, H_2_O, rt, 2 h; (**c**) for lysine-OMe-mangostin: H-Lys(Fmoc)-OMe·HCl, DIC, HOBt, anhydrous DMF, rt, overnight; then piperidine, DMF, rt, 20 min; (**d**) for 3–7: corresponding basic amino acid, DIC, HOBt, anhydrous DMF, rt, overnight. R in order: (**2**) L-Lysine-OMe; (**3**) L-Histidine-OMe; (**4**) L-Arginine-OMe; (**5**) L-Arginine-OEt; (**6**) L-Arginine-NH_2_; (**7**) L-Arginine-OtBu.

The two phenolic groups in the C3 and C6 positions of α-mangostin were functionalised in order to create a novel series of α-mangostin analogues through their chemical modification with basic amino acid residues. The possibility that an intramolecular hydrogen bond will form between the C1 hydroxyl group and the C9 carbonyl group makes the C1 hydroxyl group less reactive. The appropriate esters were created through a first alkylation using methyl bromoacetate. To create acid-ester mangostin, the esters were subsequently hydrolysed with lithium hydroxide. Thereafter, the conjugates were created by conjugating it with the amino acids of lysine, arginine or histidine using the coupling agents of *N, N′*-diisopropylcarbodiimide (DIC) and N-hydroxybenzotriazole (HOBt) in anhydrous DMF at room temperature. In a two-step method, acid ester mangostin was reacted with (Fmoc)-lysine-OMe. HCl was applied in the presence of DIC and HOBt, and the Fmoc protecting group was then removed using piperidine in DMF. The result was NH_2_-lysine-OMe-mangostin (Scheme 18).

**Figure 25:**
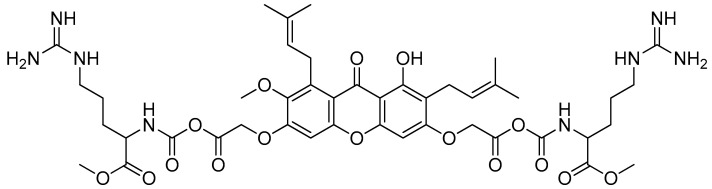
Arginine-OMe-mangostin.

Arginine-OMe-mangostin (Figure 25) showed promising antibacterial properties (Table 13). The arginine–mangostin conjugate, with an MIC value of 2 µg/mL, revealed the strongest antibacterial activity against *S. aureus* and *B. cereus* among all the derivatives, comparable to α-mangostin. The other conjugates, including lysine–mangostin and histidine–mangostin, were less effective than α-mangostin. This is most likely because cationic moieties are important for the creation of amphiphilic structures. Cationic moieties are also essential for electrostatic interactions, providing quick access to the cytoplasmic membrane. Positively charged residues, especially arginine, make it easier for peptides to enter cells. A stiff hydrophobic core of α-mangostin with two or more aromatic rings is also an important requirement for the activity. α-Mangostin can rupture the membrane bilayer, but only to a limited extent, due to its hydrophobic core and cationic moieties. In order to provide the straightforward access required for the bulky xanthone to penetrate the cytoplasmic membrane, a lipophilic chain in the form of an isoprenyl group or in the reduced form of an isoprenyl group is required [71].

The insertion of cationic amino acids and lipophilic chains into the hydrophobic core of α-mangostin resulted in conjugates with superior biological properties. The rationale behind the design could lead to the discovery of novel antimicrobials which could be useful for tackling the rising issue of antibiotic resistance. [71].

## 3. Materials and Methods

The articles used in the preparation of this review were collected through a thorough search in journal indexing databases, including Scopus, PubMed and Google Scholar. The keywords used were “natural compound conjugated amino acid”, “conjugated amino acid synthesis” and “natural compound amino acid synthesis”.

The inclusion criteria were as follows: (1) research articles, (2) chemical synthesis research and (3) papers that described the utilization of natural compounds conjugated with amino acids. In the first stage of the article collection, 1045 journal articles met the outlined criteria. However, after further examination, only 132 journal articles were chosen to be used for this review.

## 4. Conclusions: Future and Prospect

The conjugation of certain amino acids with natural compounds offers opportunities for the creation of bioactive molecules with better/equivalent pharmacological effects than/to those of the parent compounds. Conjugation was expected to change not only the pharmacological effect but also the pharmacokinetic aspect of the parent compound. Given the fact that amino acids are found in almost all parts of living things, it was expected that the insertion of amino acids into the natural compounds would facilitate the interaction with the desired living target.

Structurally, amino acids have two functional groups, with additional reactive groups in the sidechain. The functional groups provide a wide range of possibilities for amino acid conjugations with natural compounds, as discussed in this review. The conjugation may create a new compound through an ester or amide linkage, with the sidechain being available for another conjugation. The conjugates can also be designed by combining natural compounds not only with amino acids but also with di-, tri- or oligo-peptides to expand the possibility of creating new products with a better efficacy and more acceptable physicochemical properties.

Furthermore, several amino acids, such valine and glycine, showed a good bioactivity. It is not impossible that a conjugation with these amino acids can improve or enhance the pharmacological properties of the natural compound. However, a more in-depth investigation is required. A conjugate of piperine and valine revealed a high efficiency against amastigotes that was explained by the increased need of valine for metabolism by the *L. donovani* parasite.

Natural compounds conjugated with amino acids have shown excellent potential as new active compounds. A variety of synthesis processes for the conjugation of natural substances with amino acids may be a notable point of interest for researchers. Generally, the conjugation between natural products and amino acids can be achieved via the formation of an ester bond or an amide linkage. This approach can be achieved because of the amino acids’ possession of two reactive functional groups in the structure. In the formation of an ester bond from the carboxylic group, the free amino group may be able to act as an active group against the target protein, and vice versa. The strategies used to create the ester or amide analogues were mostly successful in preparing analogues with better biological properties. Although there are several conjugates that do not produce an excellent biological performance, the analogues can still be used as a reference in the consideration and selection of amino acids for successful modifications in the future.

## Figures and Tables

**Table 1:** Antileishmanial activity and cytotoxicity of piperoyl–amino acid conjugates. (**1**) Piperine; (**2**) piperic acid; (**3**) piperoyl-L-phenylalanine methyl ester; (**4**) piperoyl-L-tyrosine methyl ester; (**5**) piperoyl-L-valine methyl ester; (**6**) piperoyl-L-methionine methyl ester; (**7**) piperoyl-L-tryptophan methyl ester; (**8**) piperoyl-L-phenylalanine; (**9**) piperoyl-L-tyrosine; (**10**) piperoyl-L-valine; (**11**) piperoyl-L-methionine; (**12**) piperoyl-L-tryptophan; (**13**) tetrahydropiperoyl-L-phenylalanine methyl ester; (**14**) tetrahydropiperoyl-L-tyrosine methyl ester; (**15**) tetrahydropiperoyl-L-valine methyl ester; (**16**) tetrahydropiperoyl-L-methionine methyl ester; (**17**) tetrahydropiperoyl-L-tryptophan methyl ester.

Compound	IC_50_ (mM)	
	Amastigotes	Promastigotes
**1**	0.75	2.56
**2**	0.39	1.76
**3**	0.19	0.83
**4**	0.21	0.79
**5**	0.07	0.88
**6**	0.17	0.82
**7**	0.12	0.86
**8**	0.26	0.93
**9**	0.25	0.76
**10**	0.31	1.03
**11**	0.24	1.31
**12**	0.24	1.03
**13**	0.21	0.67
**14**	0.20	0.54
**15**	0.24	0.72
**16**	0.22	0.61
**17**	0.18	0.47

**Table 2:** Effects of poly-R-(L-glutamic acid) conjugates of camptothecin on the in vivo growth of subcutaneous B-16 melanomas. TGD: tumour growth delays. (**1**) Poly-R-(L-glutamic acid)-camptothecin; (**2**) poly-R-(L-glutamic acid)-glycine-camptothecin; (**3**) poly-R-(L-glutamic acid)-alanine-camptothecin; (**4**) poly-R-(L-glutamic acid)-(β-alanine)-camptothecin; (**5**) poly-R-(L-glutamic acid)-(4-NH-butyryl)-camptothecin; (**6**) poly-R-(L-glutamic acid)-(2-O-acetyl)-camptothecin; (**7**) poly-R-(L-glutamic acid)-(4-O-butyryl)-camptothecin; (**8**) poly-R-(L-glutamic acid)-(γ-glu)-camptothecin.

Compound	Dose (mg)	B-16 TGD (Days)
**1**	48	4
**2**	35	2
**3**	62	2
**4**	67	1
**5**	60	1
**6**	75	4
**7**	35	3
**8**	41	1

**Table 3:** In vitro antimalarial activities of compounds against the chloroquine-sensitive 3D7 strain of *Plasmodium falciparum*. (**1**) Quinine; (**2**) boc-glycine-quinine; (**3**) boc-L-alanine-quinine; (**4**) boc-L-phenylalanine-quinine; (**5**) boc-L-isoleucine-quinine; (**6**) boc-L-histidine-quinine; (**7**) boc-L-serine-quinine; (**8**) boc-L-glutamic acid-quinine; (**9**) Z-L-lysine-quinine; (**10**) Z-L-aspartic acid-quinine; (**11**) Z-L-cysteine-quinine; (**12**) Z-L-alanine-L-phenylalanine-quinine; (**13**) Z-L-valine-L-leucine-quinine; (**14**) Z-L-isoleucine-glycine-quinine.

Compound	IC_50_ (nM)
**1**	18
**2**	27
**3**	36
**4**	38
**5**	550
**6**	42
**7**	95
**8**	76
**9**	71
**10**	17
**11**	40
**12**	120
**13**	74
**14**	23

**Table 4:** Solubility of quercetin and quercetin–amino acid conjugates in PBS buffer. (**1**) Quercetin; (**2**) quercetin-alanine; (**3**) quercetin-valine; (**4**) quercetin-lysine; (**5**) quercetin-phenylalanine; (**6**) quercetin-aspartic acid; (**7**) quercetin-methionine; (**8**) quercetin-glutamic acid; (**9**) quercetin-alanine-aspartic acid; (**10**) quercetin-alanine-glutamic acid.

Compound	Water Solubility (μM)	Fold Increase
**1**	50	1.0
**2**	1290	25.8
**3**	767	15.3
**4**	338	6.8
**5**	1766	35.3
**6**	2262	45.2
**7**	675	13.5
**8**	2649	53.0
**9**	2093	41.9
**10**	2628	52.6

**Table 5:** In vitro antibacterial activity of amino-acid-modified flavone compounds. (**1**) Icaritin; (**2**) icaritin-L-histidine; (**3**) icaritin-L-arginine; (**4**) icaritin-L-lysine.

Compound	Bacterial Strains, µg/mL (µM)	
	*S. aureus*	*B. cereus*
**1**	50	50
**2**	>50	>50
**3**	1.56	3.13
**4**	25	25

**Table 6:** Antibacterial activity of curcumin bioconjugates (values represent the zone of inhibition in mm) against multi-resistant bacterial strains. (-) Resistant. (**1**) curcumin; (**2**) 4,40-(di-O-glutamoyl)-curcumin; (**3**) 4,40-(di-O-valinoyl) curcumin; (**4**) 4,40-(di-O-glycinoyl) curcumin; (**5**) monoglycinoyl curcumin; (**6**) monovalinoyl curcumin.

Bacteria	Compound					
	1	2	3	4	5	6
	Inhibition Zone (mm)					
*Micrococci*	-	18	16	15	-	26
*K. aeruginosa*	-	9	-	-	18	18
*S. saprophyticus*	-	-	14	14	-	-
*E. cloacae*	-	-	20	12	-	25
*E. coli*	10	-	20	-	12	13

**Table 7:** MICs of THC–amino acid conjugates against Gram-positive and Gram-negative bacteria. (**1**) THC, (**2**) THC-L-alanine, (**3**) THC-L-isoleucine, (**4**) THC-L-proline, (**5**) THC-L-valine, (**6**) THC-L-phenylalanine, (**7**) THC-L-glycine, and (**8**) THC-L-leucine.

Compound	MIC (µM)			
	*B. cereus*	*S. aureus*	*E. coli*	*Y. enterocolitica*
**1**	1066	1329	1723	2114
**2**	340	437	583	777
**3**	334	459	543	585
**4**	309	353	485	618
**5**	263	482	526	657
**6**	337	487	600	750
**7**	257	360	514	617
**8**	334	376	501	585

**Table 8:** IC_50_ evaluated after 72 h incubation in A431 cancer cell lines and human keratinocytes as the control. (**1**) Betulin; (**2**) betulinic acid; (**3**) betulin-lysine; (**4**) betulin-ornithine.

Compound	IC_50_ (μM)	
	HaCat Cells, 72 h	A431 Cells, 72 h
**1**	150.2	45.2
**2**	160.2	35.6
**3**	145.2	2.3
**4**	146.3	4.5

**Table 9:** The in vitro cytotoxicity of glycyrrhetinic acid conjugates with esters in the C-3 position against cancer cell lines. (**1**) Glycyrrhetinic acid; (**2**) cisplatin; (**3**) glycyrrhetinic acid-L-glycine; (**4**) glycyrrhetinic acid-L-alanine; (**5**) glycyrrhetinic acid-L-phenylalanine; (**6**) glycyrrhetinic acid-L-proline; (**7**) glycyrrhetinic acid-L-sarine; (**8**) glycyrrhetinic acid-L-leucine; (**9**) glycyrrhetinic acid-L-isoleucine; (**10**) glycyrrhetinic acid-L-methionine.

Compound	IC_50_ (µM)	
	A549	MCF7
**1**	>40	>40
**2**	9.0	6.8
**3**	2.8	3.8
**4**	3.1	4.3
**5**	16.9	14.4
**6**	4.7	5.2
**7**	4.9	3.8
**8**	22.9	11.9
**9**	4.9	10.0
**10**	7.5	8.0

**Table 10:** The in vitro cytotoxicity of glycyrrhetinic acid conjugates with amide linkages in the C-3 position against cancer cell lines. (**1**) Glycyrrhetinic acid; (**2**) cisplatin; (**3**) glycyrrhetinic acid-L-glycine; (**4**) glycyrrhetinic acid-L-alanine; (**5**) glycyrrhetinic acid-L-phenylalanine; (**6**) glycyrrhetinic acid-L-proline; (**7**) glycyrrhetinic acid-L-sarine; (**8**) glycyrrhetinic acid-L-leucine; (**9**) glycyrrhetinic acid-L-isoleucine; (**10**) glycyrrhetinic acid-L-methionine.

Compound	IC_50_ (µM)	
	A549	MCF7
**1**	>40	>40
**2**	9.0	6.8
**3**	2.4	2.8
**4**	2.1	2.1
**5**	3.0	3.3
**6**	3.3	7.6
**7**	3.3	3.4
**8**	3.3	6.1
**9**	3.4	4.7
**10**	6.9	7.0

**Table 11:** Effects of arctigenin and its amino acid derivatives on tumour growth in H22 tumour-bearing mice. (**1**) Cyclophosphamide; (**2**) arctigenin; (**3**) arctigenin-L-methionine; (**4**) arctigenin-L-phenylalanine; (**5**) arctigenin-L-valine.

Compound	Dosage (mg/kg)	Inhibitory Rate (%)
**1**	25	72.06
**2**	40	26.26
**3**	40	55.87
**4**	40	51.40
**5**	40	69.27

**Table 12:** Cytotoxic activity of podophyllotoxin–amino acid conjugates in vitro (IC_50_, nM). (**1**) Podophyllotoxin-Boc-L-glycine; (**2**) podophyllotoxin-Boc-L-sarcosine; (**3**) podophyllotoxin-Boc-L-alanine; (**4**) podophyllotoxin-Boc-L-phenylalanine; (**5**) podophyllotoxin-L-glycine; (**6**) podophyllotoxin-L-sarcosine; (**7**) podophyllotoxin-L-alanine; (**8**) podophyllotoxin-L-phenylalanine; (**9**) doxorubicin.

Compound	IC_50_ (nM)			
	A549	MCF-7	HepG2	L-02
**1**	1.6	11.3	26.6	7.3
**2**	9.5	132.6	96.4	160.2
**3**	17.3	106.9	81.5	109.5
**4**	30.2	95.3	87.1	78.4
**5**	12.9	7.2	17.5	14.5
**6**	6.8	17.5	24.9	9.6
**7**	13.6	18.5	17.8	10.8
**8**	3.8	15.6	27.3	8.9
**9**	228.2	75.6	693.1	53.3

**Table 13:** In vitro antibacterial activity of amino-acid-modified α-mangostin. (**1**) α-Mangostin; (**2**) α-mangostin-OMe-lysine; (**3**) α-mangostin-OMe-histidine; (**4**) α-mangostin-OMe-arginine; (**5**) α-mangostin-OEt-arginine; (**6**) α-mangostin-NH2-arginine; (**7**) α-mangostin-OtBu-arginine.

Compound	MIC Values, µg/mL (µM)	
	*S. aureus*	*B. cereus*
**1**	2	2
**2**	6	12
**3**	>50	>50
**4**	2	2
**5**	6	12
**6**	6	6
**7**	12	12

## Data Availability

Not applicable.
